# Trends and predictors of extreme preterm birth: Western Australian population-based cohort study

**DOI:** 10.1371/journal.pone.0214445

**Published:** 2019-03-26

**Authors:** Brad M. Farrant, Scott W. White, Carrington C. J. Shepherd

**Affiliations:** 1 Telethon Kids Institute, The University of Western Australia, West Perth, Western Australia, Australia; 2 Division of Obstetrics and Gynaecology (M550), The University of Western Australia, Crawley, Western Australia, Australia; 3 Department of Maternal Fetal Medicine, King Edward Memorial Hospital, Subiaco, Western Australia, Australia; University of Calgary, CANADA

## Abstract

**Background:**

The preterm birth rate is rising in high-income countries and is associated with increased mortality and morbidity. Although the risks increase with greater prematurity and risk factors have been found to vary with gestational age and labour onset, few studies have focused on the myriad pathways to extreme preterm birth (20–27 weeks’ gestation). The current study investigated trends in extreme preterm birth by labour onset type and examined the antecedent risks to further our understanding around the identification of high-risk pregnancies.

**Methods:**

Retrospective cohort study including all singleton extreme preterm births in Western Australia between 1986 and 2010. De-identified data from six core population health datasets were linked and used to ascertain extreme preterm births (excluding medical terminations and birth defects) after spontaneous onset of labour, preterm pre-labour rupture of membranes, and medically indicated labour onset. Trends over time in extreme preterm birth were analysed using linear regression. Multivariable regression techniques were used to assess the relative risks associated with each salient, independent risk factor and to calculate Population Attributable Risks (PARs).

**Results:**

The extreme preterm birth rate including medical terminations and birth defects significantly increased over time whereas the extreme preterm birth rate excluding medical terminations and birth defects did not change. After medical terminations and birth defects were excluded, the rate of medically indicated extreme preterm births significantly increased over time whereas the rate of preterm pre-labour rupture of membranes extreme preterm births significantly reduced, and the rate of spontaneous extreme preterm births did not significantly change. In the multivariate analyses, factors associated with placental dysfunction accounted for >10% of the population attributable risk within each labour onset type.

**Conclusions:**

First study to show that the increase in extreme preterm birth in high-income jurisdiction is no longer evident after medical terminations and birth defects are excluded. Interventions that identify and target women at risk of placental dysfunction presents the greatest opportunity to reduce extreme preterm births.

## Introduction

Research has demonstrated that the preterm birth (< 37 weeks gestation) rate is rising in high-income countries [1–4]. Preterm birth is associated with marked increases in mortality and morbidity in the perinatal period, adverse outcomes throughout the lifecourse, and is the pre-eminent problem facing obstetricians and neonatologists in high-income countries [1]. Typically, there is an increase in health and developmental risks with greater prematurity—for example, the stillbirth rate for singleton births in Western Australia from 1986–2010 was 523.0 per 1,000 for births at 20–27 weeks gestation compared with 19.6 per 1,000 for preterm births at 32–36 weeks [5].

A complex and inter-related range of risk factors for preterm birth have been identified in the extant literature. These include distal characteristics associated with sociodemographic circumstances, genetic traits, reproductive history and some maternal medical conditions, and factors that are more proximal to the birth—such as complications of pregnancy and delivery [6–9]. An emerging body of research indicates that the predictors of preterm birth differ with the degree of prematurity, although few studies have focused specifically on the profile of risk in early gestational epochs, when health outcomes are most likely to be compromised [10–12]. Given that perinatal mortality is most commonly experienced in the extreme preterm period, a focus on antecedent risks in this period may provide the greatest opportunities to support obstetric and public health interventions aimed at reducing perinatal loss.

Antecedent risk profiles have been shown to differ by labour onset types, reflecting the myriad different pathways to preterm birth. The evidence-base, to-date, is derived from a handful of studies that have investigated the trends and predictors of all preterm births after spontaneous onset of labour, pre-labour rupture of membranes, and medically indicated labour onset [1, 8, 9, 13–16]. None of these studies has focused on the predictors of extreme preterm births in particular. The aim of the current study was, therefore, to: (1) use total population linked health data in Western Australia to provide a more nuanced understanding of the antecedent risks to births at 20–27 weeks gestation, and how this varies by labour onset type, and (2) by doing so, to further our understanding around the identification of high-risk pregnancies and, where appropriate, identify targets for intervention that have the potential to decrease the incidence of extreme preterm birth and associated mortality and morbidity. In our previous research we found that, when assessing changes in rates over time, it is important to account for birth defects and late pregnancy termination (20 weeks or more gestation) [5]. Accordingly, in the current study we investigated trends in the extreme preterm birth rate including and excluding medical terminations and birth defects.

## Methods

### Study population and data sources

This retrospective cohort study included all singleton term and extreme preterm births in Western Australia between 1986 and 2010, inclusive. Study data were sourced from core population health datasets held by the Data Linkage Branch of the Western Australian Government Department of Health (DLB). These datasets include the Midwives’ Notification System (MNS), Western Australian Register of Developmental Anomalies (WARDA), the Birth Registration and Death Registration datasets (from the Western Australian Registry of Births, Deaths and Marriages), the Hospital Morbidity Data Collection (HMDC), and the Mental Health Information System (MHIS).

The MNS records the circumstances of all births of 20 weeks or more gestation, with information received from attending midwives. The WARDA includes all birth defects diagnosed at birth for stillbirths (including terminations of pregnancy) and livebirths as well as diagnoses for livebirths up to six years of age from a number of sources with a high level of ascertainment [17]. The HMDC collects information on discharges from all hospitals (public and private) in Western Australia and the MHIS records information on mental health outpatient admissions.

These data were linked together by the DLB by probabilistic linkage using common identifiers including name, address and birthdate [18]. Multiple linkage passes are conducted in order to minimise both false-positive and false-negative errors along with clerical review to resolve doubtful links. The procedures used in the extraction of data from the WA Data Linkage System (WADLS) have been internationally accepted as best practice [19] and the quality of linkages have been shown to be highly reliable [18]. The MNS record was used as the initial master file and data linked across datasets. The MNS has been found to have greater than 99% ascertainment of births in WA from 1980 onwards [20] and over 97% agreement with corresponding medical records regarding pregnancy complications and pre-existing medical conditions [21]. Only de-identified data files were extracted (for each dataset) by the DLB and provided to the researchers [22]. We then merged the datasets using a linkage key.

This research was granted ethics approval by the Western Australian Department of Health Human Research Ethics Committee (#2011/64) and the Western Australian Aboriginal Health Ethics Committee (#613). These ethical approvals support a waiver of consent on the basis that the study: (1) utilises routinely collected information from existing administrative datasets (and, accordingly, does not include active participants); and (2) only has access to de-identified data, which are stored, analysed and disseminated according to strict protocols.

### Extreme preterm birth

Consistent with World Health Organization guidelines [23], extreme preterm birth was defined as occurring between 20 and 27 weeks gestation (inclusive) and term as 37 or more weeks gestation. Based on data on labour onset and pre-labour rupture of membranes, extreme preterm births were subdivided into three labour onset categories: Spontaneous if labour onset occurred spontaneously with intact membranes, preterm pre-labour rupture of membranes if spontaneous rupture of membranes occurred prior to the onset of labour, and medically indicated where labour was induced or caesarean section occurred prior to labour or preterm pre-labour rupture of membranes.

### Risk factors

We included data on a range of known and available risk factors that typically occur prior to 20 weeks gestation, including sociodemographic, reproductive history, maternal conditions, and pregnancy complications. Sociodemographic data included socio-economic status, geographic isolation, maternal age, marital status, maternal ethnicity and sex of the child. Socio-economic status at the time of the birth was assessed using the Socio-Economic Index for Areas (SEIFA) index of relative socio-economic disadvantage. The SEIFA index is calculated by the Australian Bureau of Statistics based on population census data relating to householder’s education, occupation, employment, income, housing and household composition [24]. The level of mother’s relative geographic isolation was assessed using the Accessibility/Remoteness Index of Australia based on the mother’s usual residence at the time of the child’s birth. The Level of Relative Isolation has five categories that range from none (Perth metropolitan area) through to extreme.

Maternal ethnicity was sourced from the MNS. Maternal self-report of ethnic origin has been categorised by the attending midwife into the categories of Caucasian, Aboriginal and/or Torres Strait Islander (Indigenous Australian), Asian (including Chinese, Japanese, Vietnamese, Cambodian and other South-East Asian origins), Indian (Indian subcontinent), African, Polynesian and Maori. The number of births to Polynesian women was very small and, accordingly, the maternal ethnicity of these cases was recoded and included in the group ‘Other’ which includes ethnicities not covered by the above [25]. Maternal age, marital status, and sex of the child were also sourced from the MNS.

Maternal reproductive history data sourced from the MNS included the number of previous pregnancies, stillbirth(s), preterm birth(s), and caesarean section(s). Maternal conditions included essential hypertension, maternal asthma, genital herpes, smoking, pre-existing diabetes mellitus, overweight/obesity, substance abuse, and mental health diagnosis. Data relating to essential hypertension, maternal asthma, genital herpes, and smoking cigarettes during pregnancy were sourced from the MNS. Maternal overweight/obesity diagnosis (principal- or co-diagnosis) prior to the birth of the child was derived from the HMDC records when any of the following ICD diagnostic codes were recorded: ICD-10 E56 and E68.9, ICD-9 278, and ICD-8 277 and 278. Maternal substance abuse diagnosis (principal- or co-diagnosis) prior to the birth of the child was derived from the HMDC and MHIS records when any of the following ICD diagnostic codes were recorded: ICD-10 F10 to F19 and F55, ICD-9 291, 292, and 303 to 305, or ICD-8 291, 294.3, 303 and 304. Maternal mental health diagnosis (principal- or co-diagnosis) prior to the birth of the child was derived from the HMDC and MHIS records when any of the following ICD diagnostic codes were recorded: ICD-10 F00 to F09, F20 to F54, F56 to F99, and X60 to X84, ICD-9 290, 293 to 302, 306 to 319, and E950 to E959, or ICD-8 290, 292 to 294.29, 294.4 to 302, 305 to 319, and E950 to E958. ICD diagnostic codes were selected to be as inclusive as possible but it should be noted that, as they relate to hospital admissions, they likely reflect only the most serious cases of maternal overweight/obesity, maternal substance abuse, and maternal mental health problems.

Pregnancy complications data sourced from the MNS included threatened abortions, urinary tract infection, pre-eclampsia, antepartum haemorrhage (placenta praevia, placental abruption, other), threatened preterm labour, fertility treatment, chorionic villus sampling/placental biopsy, amniocentesis, and year of birth. MNS data regarding gestational age and birthweight were used to calculate a measure of growth restriction: growth/weight for gestational age using Australian weight for gestational age norms [26].

### Terminations of pregnancy

Information on pregnancy terminations at 20 weeks or more gestation was obtained from the WARDA and the cause of death text field on the Death Registration record. In Western Australia, all termination procedures are notifiable under the Health Act 1911. While no upper gestational age limit is specified in the legislation, the vast majority of terminations are conducted prior to 20 weeks [27]. Late terminations (≥ 20 weeks) require approval by a panel of medical practitioners and are restricted to cases where there is a serious medical condition affecting the mother or fetus.[28] Terminations at this gestation are generally referred to as ‘late’ terminations and are managed and conducted in a structured manner in accordance with legislation [27].

### Birth defects

The WARDA was used to identify children with birth defects, and codes cases according to the British Paediatric Association extension of the International Classification of Diseases Version 9. All cases with a birth defect diagnosis code(s) were classified as having a birth defect for the purposes of this study. A full list of birth defects diagnosis codes is available on the WARDA website [29].

### Statistical analysis

Trends over time in the extreme preterm birth rate were analysed using linear regression, with year of birth specified as a (continuous) classification variable and the t-statistic used to assess statistical significance (for ease of comparison, standardised regression coefficients (*β*s) are reported along with absolute changes in rates (based on unstandardised regression coefficients) where significant changes are observed). The assumptions for linear regression were assessed by visual inspection of scatterplots for linearity and by using normal probability plots of the standardised residuals along with scatterplots of the standardised predicted value versus the standardised residuals.

Univariable and multivariable logistic regression was used to assess the relative risk of spontaneous, preterm pre-labour rupture of membranes and medically indicated extreme preterm birth associated with each risk factor in comparison with spontaneous term birth (risk ratios reported) after excluding medical terminations and cases with birth defects (spontaneous term births were used as the comparison group because they represent the preferred outcome). Risk factors that had a significant risk ratio at the univariable level were included in the relevant multivariable model, with the exception of birth year group, which was included in all models to control for changes in the effects of risk factors over time. Adjusted estimates of relative risk were derived using a modified Poisson regression model (Generalized Linear Model) with a robust error variance [30]. For each risk factor that had a significant risk ratio at the multivariable level, population attributable risks (PARs) were derived using the adjusted risk ratio. We report PARs with 95% confidence intervals (95% CI) which were calculated using the method described by Hildebrandt et al. [31]. All analyses were conducted using SPSS v23 and SAS v9.4 (SAS Institute Inc., Cary, NC, USA, 2016). The alpha level was set at .05 for all analyses.

## Results

### Extreme preterm birth rate 1986–2010

**Fig 1:**
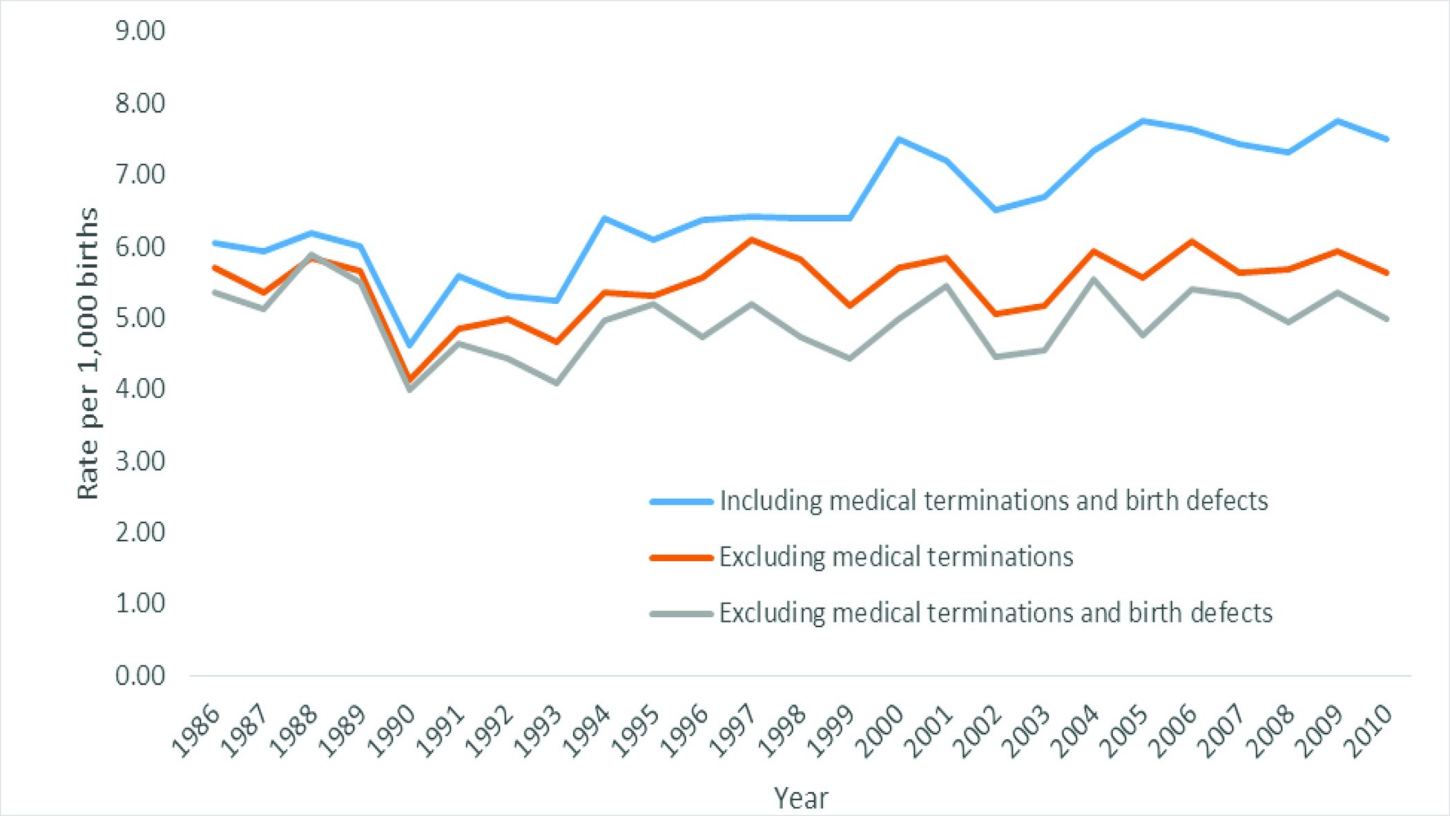
Extreme preterm birth rate including and excluding medical terminations and birth defects.

For the 1986–2010 period there were 638,463 singleton births (327,369 male, 311,056 female, 38 undetermined) including 4,202 extreme preterm (20–27 weeks gestation) births (6.6 extreme preterm births per 1,000 births). After excluding medical terminations, there were 637,696 singleton births (326,975 male, 310,691 female, 30 undetermined) including 3,493 extreme preterm births (5.5 extreme preterm births per 1,000 births). After excluding medical terminations and cases with birth defects (minor and major), for the 1986–2010 period there were 603,351 singleton births (306,994 male, 296,337 female, 20 undetermined) including 2,997 extreme preterm births (5.0 extreme preterm births per 1,000 births). As can be seen in Fig 1, the extreme preterm birth rate including medical terminations and birth defects significantly increased over time (*β* = 0.84, *p* < .001; an increase of 0.10 per 1,000 births per year), the extreme preterm birth rate excluding medical terminations demonstrated a non-significantly increase over time (*β* = 0.39, *p* = .06), and the extreme preterm birth rate excluding medical terminations and birth defects did not change over time (*β* = 0.08, *p* = .72).

**Fig 2:**
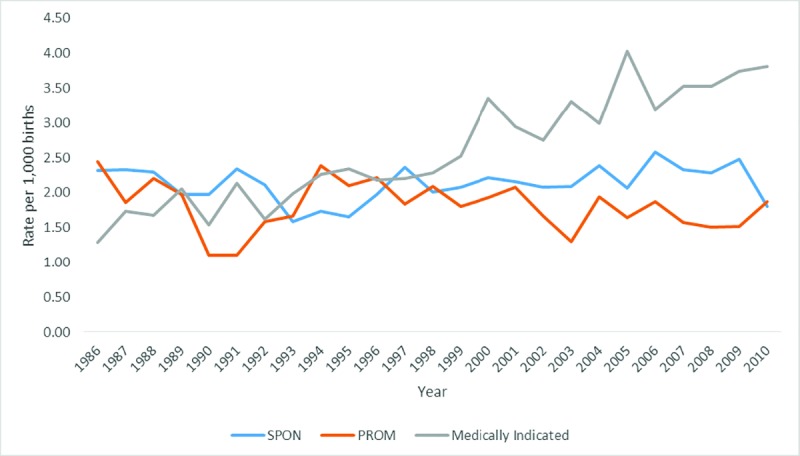
Prevalence of extreme preterm births by birth type including medical terminations and birth defects.

The incidence of spontaneous, preterm pre-labour rupture of membranes and medically indicated extreme preterm births including medical terminations and birth defects are displayed in Fig 2. The rate of medically indicated MI extreme preterm births significantly increased over time (*β* = 0.94, *p* < .001; an increase of 0.10 per 1,000 births per year) whereas the rate of spontaneous extreme preterm births (*β* = 0.20, *p* = .34) and preterm pre-labour rupture of membranes extreme preterm births (*β* = -0.23, *p* = .26) did not significantly change over time.

**Fig 3:**
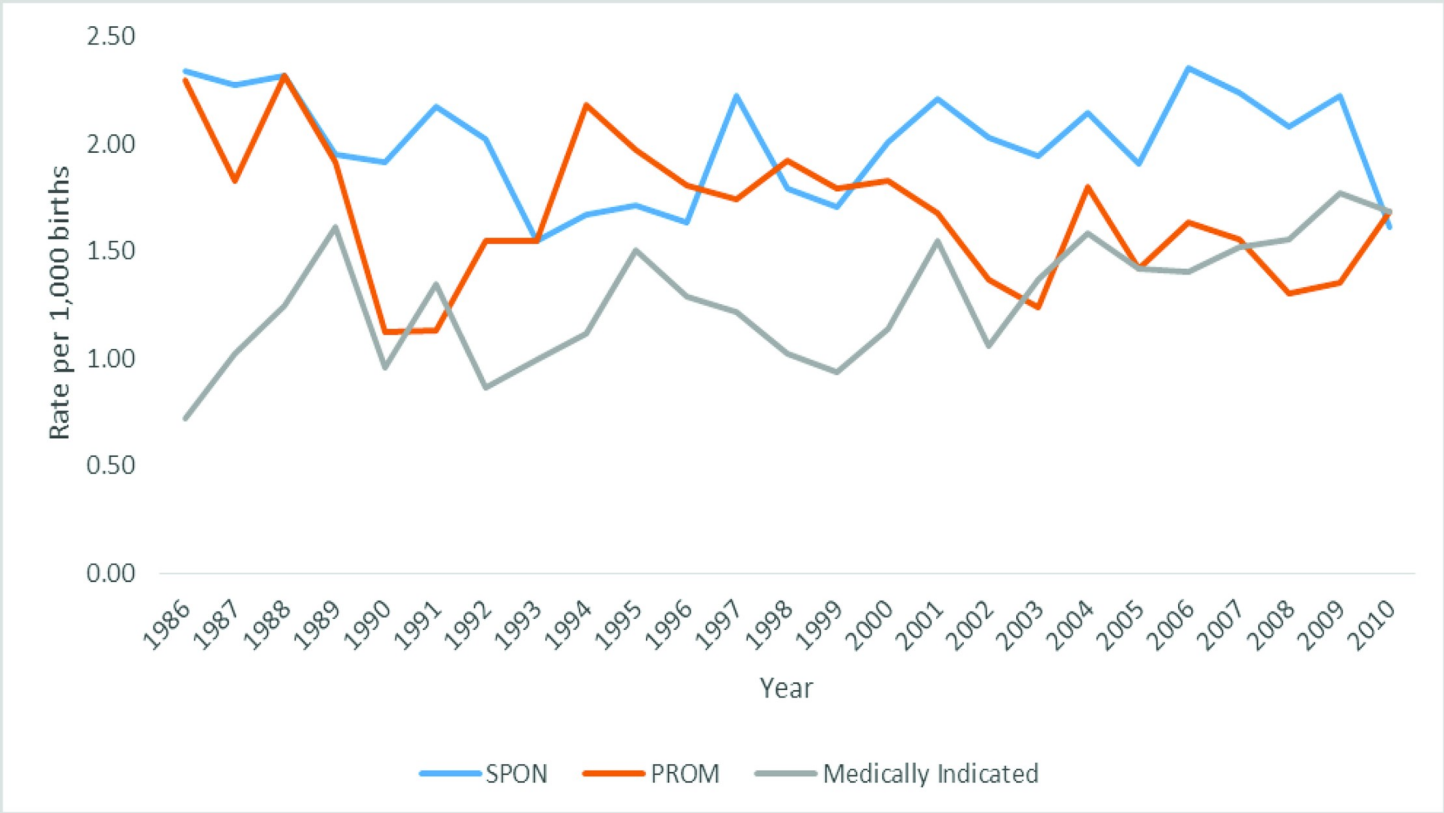
Prevalence of extreme preterm births by birth type excluding medical terminations and birth defects.

Fig 3 displays the incidence of spontaneous, preterm pre-labour rupture of membranes and medically indicated extreme preterm births after medical terminations and birth defects are excluded. The rate of medically indicated extreme preterm births significantly increased over time (*β* = 0.63, *p* = .001; an increase of 0.02 per 1,000 births per year). Thus, the majority of the increase (around 80%) in the rate of medically indicated extremely preterm births is explained by medical terminations and birth defects (for a breakdown of the change in the rate of risk factors over time see S1 Table). In contrast, the rate of preterm pre-labour rupture of membranes extreme preterm births fluctuates over the study period but demonstrated a significant reduction between 1986 and 2010 (*β* = -0.41, *p* = .04; a decrease of 0.02 per 1,000 births per year; see Fig 3). The rate of spontaneous extreme preterm births did not significantly change over time (*β* = -0.04, *p* = .87).

### Risk factor analysis 1998–2010

Data on the full range of risk factors was available for 1998–2010 (3.7% of cases had missing data on one or more variables). For this period, excluding medical terminations and cases with birth defects, complete sets of data were available for 629 cases of spontaneous extreme preterm birth, 496 cases of extreme preterm birth with preterm pre-labour rupture of membranes, and 430 cases of medically indicated extreme preterm birth. Risks are compared to those for spontaneous term births (*N* = 150,584). Descriptive statistics (% of births) and the results of univariate logistic regression analyses are shown in Table 1.

**Table 1:** Univariate analysis of risk factors for extreme preterm birth excluding medical terminations and birth defects 1998–2010.

	Spontaneous term Births (*N* = 150,584)	Spontaneous extreme preterm (*N* = 629)		Preterm pre-labour rupture of membranes extreme preterm (*N* = 496)		Medically indicated extreme preterm (*N* = 430)	
	% of births	% of births	Unadjusted Risk Ratio^a^ (95% CI)	% of births	Unadjusted Risk Ratio^a^ (95% CI)	% of births	Unadjusted Risk Ratio^a^ (95% CI)
Time period 2007–2010 2003–2006 1998–2002	34.8 28.7 36.5	35.3 30.5 34.2	1.08 (0.90–1.31) 1.13 (0.93–1.38) Ref	33.1 29.0 37.9	0.92 (0.74–1.13) 0.97 (0.78–1.21) Ref	39.5 31.2 29.3	1.42* (0.12–1.78) 1.35* (1.06–1.72) Ref
**Sociodemographic**							
SES (SEIFA disadvantage—quintiles) 1 2 3 4 5	20.1 23.3 21.1 18.8 16.7	25.8 23.5 21.3 15.7 13.7	1.56^†^ (1.20–2.02) 1.23 (0.94–1.60) 1.23 (0.94–1.61) 1.02 (0.77–1.36) Ref	25.6 20.6 21.8 16.7 15.3	1.38* (0.04–1.84) 0.96 (0.71–1.29) 1.12 (0.84–1.50) 0.97 (0.71–1.32) Ref	20.9 23.0 20.5 19.8 15.8	1.10 (0.80–1.50) 1.04 (0.76–1.42) 1.02 (0.74–1.40) 1.11 (0.81–1.53) Ref
Level of relative isolation (ARIA) Extreme High Moderate Low None	8.3 3.8 10.9 33.6 43.5	14.0 3.0 8.9 33.5 40.5	1.80^†^ (1.42–2.30) 0.86 (0.54–1.37) 0.88 (0.66–1.17) 1.07 (0.89–1.29) Ref	10.7 3.8 8.7 34.5 42.3	1.32 (0.98–1.78) 1.05 (0.66–1.67) 0.82 (0.59–1.14) 1.05 (0.86–1.29) Ref	6.7 4.9 10.0 30.7 47.7	0.74 (0.50–1.09) 1.18 (0.76–1.85) 0.84 (0.61–1.17) 0.83 (0.67–1.04) Ref
Maternal age < 20 20–24 30–34 35–39 40+ 25–29	6.8 18.9 29.1 13.1 2.1 30.0	14.5 20.0 23.4 15.9 2.5 23.7	2.68^†^ (2.07–3.48) 1.34* (1.06–1.70) 1.02 (0.81–1.27) 1.53^†^ (1.19–1.97) 1.51 (0.90–2.53) Ref	6.9 16.7 28.4 18.1 5.2 24.6	1.23 (0.84–1.80) 1.08 (0.82–1.43) 1.19 (0.93–1.51) 1.68^†^ (1.28–2.20) 2.99^†^ (1.96–4.56) Ref	7.7 15.8 30.0 16.7 5.8 24.0	1.41 (0.96–2.09) 1.05 (0.77–1.42) 1.29 (0.99–1.67) 1.60* (1.18–2.16) 3.41^†^ (2.20–5.26) Ref
Marital status No Married (including de facto)	11.2 88.8	20.3 79.7	2.02^†^ (1.67–2.45) Ref	16.9 83.1	1.62^†^ (1.28–2.04) Ref	17.9 82.1	1.73^†^ (1.35–2.21) Ref
Maternal ethnicity Indigenous Asian Indian African Maori ‘Other’ Caucasian	7.4 6.9 1.1 1.1 1.1 3.6 78.8	20.0 4.5 1.6 1.6 0.6 5.9 65.8	3.20^†^ (2.62–3.90) 0.78 (0.53–1.14) 1.66 (0.89–3.10) 1.70 (0.91–3.19) 0.71 (0.27–1.90) 1.98^†^ (1.41–2.76) Ref	17.5 4.6 1.0 2.0 1.8 4.4 68.5	2.70^†^ (2.13–3.41) 0.78 (0.51–1.19) 1.01 (0.42–2.44) 2.07* (1.11–3.88) 1.94* (1.00–3.75) 1.43 (0.93–2.20) Ref	12.8 4.4 0.7 2.6 1.2 4.2 74.2	1.82^†^ (1.37–2.42) 0.68 (0.43–1.09) 0.65 (0.21–2.02) 2.43* (1.33–4.42) 1.15 (0.48–2.78) 1.25 (0.78–2.01) Ref
Child sex—male	50.6	56.3	1.26* (1.07–1.47)	53.6	1.13 (0.95–1.35)	48.1	0.91 (0.75–1.09)
**Reproductive history**							
Previous stillbirth	1.0	7.5	7.83^†^ (5.84–10.49)	5.6	5.87^†^ (4.02–8.56)	7.0	7.34^†^ (5.08–10.60)
Previous preterm birth (<37 wks)	4.6	20.0	5.21^†^ (4.30–6.32)	22.0	5.88^†^ (4.76–7.26)	17.7	4.45^†^ (3.48–5.69)
Previous caesarean section	4.9	12.9	2.87^†^ (2.27–3.62)	14.9	3.40^†^ (2.66–4.35)	18.4	4.36^†^ (3.42–5.56)
Number of previous pregnancies None 1 2 3 4 or more	31.0 30.9 18.2 9.6 10.3	30.0 24.2 18.0 11.6 16.2	0.62^†^ (0.49–0.79) 0.50^†^ (0.39–0.64) 0.63^†^ (0.48–0.82) 0.77 (0.57–1.04) Ref	23.6 24.2 17.3 12.9 22.0	0.36^†^ (0.28–0.47) 0.37^†^ (0.29–0.48) 0.45^†^ (0.34–0.60) 0.63* (0.47–0.86) Ref	30.2 22.3 17.0 11.9 18.6	0.54^†^ (0.41–0.72) 0.40^†^ (0.30–0.54) 0.52^†^ (0.38–0.71) 0.69* (0.49–0.98) Ref
**Maternal conditions**							
Essential hypertension	0.5	0.6	1.32 (0.49–3.52)	1.2	2.52* (1.13–5.61)	5.6	11.84^†^ (7.90–17.76)
Maternal asthma	9.5	11.4	1.23 (0.97–1.58)	11.9	1.29 (0.98–1.69)	11.2	1.20 (0.89–1.62)
Genital herpes	1.6	0.2	0.10* (0.01–0.71)	0.8	0.51 (0.19–1.37)	1.9	1.19 (0.59–2.40)
Smoking	19.6	28.8	1.66^†^ (1.40–1.97)	25.6	1.41^†^ (1.16–1.73)	24.9	1.36* (1.09–1.69)
Pre-existing diabetes mellitus	0.1	1.3	8.49^†^ (4.28–16.85)	1.8	12.12^†^ (6.35–23.14)	2.1	14.01^†^ (7.33–26.78)
Overweight/Obesity diagnosis	0.8	2.1	2.52^†^ (1.46–4.36)	3.2	3.97^†^ (2.42–6.51)	4.9	6.09^†^ (3.94–9.41)
Substance Abuse diagnosis	4.0	8.6	2.24^†^ (1.70–2.96)	10.1	2.68^†^ (2.00–3.58)	7.4	1.92^†^ (1.34–2.76)
Mental Health diagnosis	10.7	20.2	2.10^†^ (1.73–2.55)	20.4	2.13^†^ (1.71–2.64)	19.8	2.05^†^ (1.62–2.60)
**Pregnancy complications**							
Threatened abortion (<20 wks)	3.8	11.3	3.22^†^ (2.52–4.12)	16.1	4.86^†^ (3.83–6.16)	6.5	1.77* (1.21–2.60)
Urinary tract infection	3.5	6.7	1.97^†^ (1.44–2.69)	5.6	1.65* (1.13–2.41)	1.9	0.52 (0.26–1.05)
Pre-eclampsia	1.3	1.1	0.88 (0.42–1.86)	0.2	0.16 (0.02–1.13)	26.5	26.78^†^ (21.72–33.03)
APH–placenta praevia	0.2	2.5	10.39^†^ (6.39–16.89)	2.8	11.61^†^ (6.89–19.56)	3.7	15.36^†^ (9.42–25.05)
APH–placental abruption	0.2	12.6	57.62^†^ (46.57–71.30)	7.3	35.43^†^ (25.68–48.87)	8.6	42.47^†^ (30.86–58.46)
APH–other	2.0	25.6	16.14^†^ (13.54–19.24)	26.2	16.82^†^ (13.81–20.48)	6.7	3.54^†^ (2.43–5.15)
Threatened preterm labour (<37 wks)	1.4	33.9	32.72^†^ (27.88–38.39)	18.8	15.55^†^ (12.46–19.40)	3.7	2.70^†^ (1.64–4.44)
Fertility treatment	1.5	6.0	4.31^†^ (3.11–5.96)	9.3	6.82^†^ (5.05–9.21)	3.3	2.28* (1.34–3.87)
CVS/placental biopsy	0.3	0.6	2.46 (0.93–6.57)	0.6	2.35 (0.76–7.28)	0.2	0.90 (0.13–6.42)
Amniocentesis	1.8	4.0	2.21^†^ (1.49–3.30)	7.9	4.54^†^ (3.28–6.28)	13.7	8.39^†^ (6.39–11.01)
Fetal growth Very small for gestation (<3%) Small for gestation (≥3% <10%) Large for gestation (>90% ≤97%) Very large for gestation (>97%) 10–90%	2.6 7.0 6.0 2.4 82.0	4.1 4.3 7.5 3.7 80.4	1.59* (1.07–2.36) 0.62* (0.42–0.92) 1.27 (0.95–1.72) 1.55* (1.02–2.35) Ref	2.8 4.8 6.9 1.4 84.1	1.04 (0.61–1.77) 0.67 (0.45–1.01) 1.12 (0.79–1.59) 0.57 (0.27–1.21) Ref	28.1 16.5 1.4 0.9 53.0	16.02^†^ (12.88–19.92) 3.61^†^ (2.77–4.71) 0.36* (0.16–0.81) 0.60 (0.22–1.61) Ref

*p < .05

^†^*p* < .001, Ref = reference category

^a^ Compared to spontaneous term births (*N* = 150,584)

Risk factors that were significant across all three extreme preterm birth labour onset groups at the univariate level were: previous stillbirth, previous preterm birth, previous caesarean section, increased maternal age, being unmarried, Indigenous maternal ethnicity, maternal smoking during pregnancy, pre-existing diabetes mellitus, maternal overweight/obesity, maternal substance abuse, maternal mental health diagnosis, threatened abortion, antepartum haemorrhage (APH)–placenta praevia, APH–placental abruption, APH–other, threatened preterm labour, fertility treatment, and amniocentesis. Whereas having fewer than 4 previous pregnancies was a significant protective factor, at the univariate level, against spontaneous, preterm pre-labour rupture of membranes and medically indicated extreme preterm birth.

Most other risk factors were only associated with one or two of the extreme preterm birth labour onset groups at the univariate level. Essential hypertension was a significant risk factor, at the univariate level, for preterm pre-labour rupture of membranes and medically indicated extreme preterm birth. Being in the most disadvantage SES quintile was a significant risk factor for spontaneous and preterm pre-labour rupture of membranes extreme preterm birth as was having a urinary tract infection. African maternal ethnicity was a significant risk factor for preterm pre-labour rupture of membranes and medically indicated extreme preterm birth. Being small or large for gestational age had significant impact, at the univariate level, on the risk of spontaneous and medically indicated extreme preterm birth (see Table 1). Other significant risk factors for spontaneous extreme preterm birth were living in areas of extreme isolation, maternal age under 24 years, ‘other’ maternal ethnicity, and the child being male. Whereas genital herpes was a significant protective factor against spontaneous extreme preterm birth. At the univariate level, being born later in the study period was a significant risk factor for medically indicated extreme preterm birth as was pre-eclampsia.

Each multivariable model included risk factors that achieved statistical significance in univariable analysis (see Table 2). At the multivariable level, previous caesarean section, being unmarried, threatened abortion, APH–placenta praevia, APH–placental abruption, APH–other, and amniocentesis were significant risk factors across all three extreme preterm birth labour onset groups. Maternal smoking during pregnancy and maternal substance abuse were not significant predictors of spontaneous, preterm pre-labour rupture of membranes and medically indicated extreme preterm birth in multivariable models. Indigenous, African and ‘other’ maternal ethnicity were significant risk factors for spontaneous and preterm pre-labour rupture of membranes extreme preterm birth. Threatened preterm labour was also a significant risk factor for spontaneous and preterm pre-labour rupture of membranes extreme preterm birth as was fertility treatment. Maternal overweight/obesity was a significant risk factor for preterm pre-labour rupture of membranes and medically indicated extreme preterm birth. Maternal mental health diagnosis was a significant risk factor for spontaneous and medically indicated extreme preterm birth. Other significant risk factors for spontaneous extreme preterm birth were having no previous pregnancies, child being male, and large or very large for gestational age. Whereas living in high or moderate areas of relative isolation, genital herpes, and being small for gestational age were significantly protective against spontaneous extreme preterm birth.

**Table 2:** Multivariate analysis of risk factors for extreme preterm birth excluding medical terminations and birth defects 1998–2010.

	Spontaneous extreme preterm (*N* = 629)		Preterm pre-labour rupture of membranes extreme preterm (*N* = 496)		Medically indicated extreme preterm (*N* = 430)	
	Adjusted Risk Ratio^a^ (95% CI)	PAR % (95% CI)	Adjusted Risk Ratio^a^ (95% CI)	PAR % (95% CI)	Adjusted Risk Ratio^a^ (95% CI)	PAR % (95% CI)
Time period 2007–2010 2003–2006 1998–2002	1.11 (0.90–1.35) 1.15 (0.93–1.42) Ref		0.90 (0.72–1.13) 0.96 (0.77–1.21) Ref		1.74^†^ (1.36–2.22) 1.41* (1.08–1.83) Ref	20.5 (13.4, 27.5) 10.5 (4.4, 16.7)
**Sociodemographic**						
SES disadvantage (quintiles) 1 2 3 4 5	0.99 (0.72–1.35) 1.04 (0.78–1.39) 1.07 (0.77–1.49) 0.92 (0.67–1.26) Ref		0.98 (0.71–1.35) 0.91 (0.67–1.24) 1.07 (0.79–1.44) 0.91 (0.66–1.26) Ref			
Level of relative isolation (ARIA) Extreme High Moderate Low None	1.01 (0.74–1.40) 0.57* (0.35–0.94) 0.69* (0.49–0.97) 1.05 (0.83–1.32) Ref	-1.6 (-3.0, -0.2) -3.5 (-6.0, -1.0)				
Maternal age < 20 20–24 30–34 35–39 40+ 25–29	1.36 (0.97–1.91) 1.06 (0.82–1.36) 1.01 (0.80–1.28) 1.31 (0.99–1.74) 1.29 (0.76–2.20) Ref		1.04 (0.68–1.58) 0.94 (0.71–1.26) 1.20 (0.94–1.55) 1.35* (1.01–1.79) 1.89* (1.20–2.99) Ref	4.4 (0.5, 8.3) 1.9 (-0.1, 3.9)	1.22 (0.79–1.87) 0.94 (0.68–1.30) 1.11 (0.83–1.48) 1.20 (0.87–1.66) 1.37 (0.83–2.26) Ref	
Marital status No Married (including de facto)	1.46^†^ (1.16–1.84) Ref	4.9 (1.4, 8.4)	1.48* (1.13–1.94) Ref	5.1 (1.4, 8.8)	1.62^†^ (1.23–2.13) Ref	6.5 (2.4, 10.6)
Maternal ethnicity Indigenous Asian Indian African Maori ‘Other’ Caucasian	2.33^†^ (1.77–3.06) 1.02 (0.70–1.49) 1.57 (0.76–3.24) 2.19* (1.18–4.08) 0.62 (0.22–1.76) 1.70* (1.16–2.48) Ref	9.1 (5.7, 12.4) 1.3 (0.3, 2.3) 2.4 (0.5, 4.3)	2.43^†^ (1.82–3.25) 0.86 (0.55–1.34) 1.26 (0.51–3.10) 3.00^†^ (1.60–5.63) 1.42 (0.65–3.09) 1.65* (1.07–2.54) Ref	9.7 (6.0, 13.3) 2.2 (0.9, 3.4) 2.3 (0.4, 4.1)	0.96 (0.66–1.39) 0.74 (0.46–1.17) 0.59 (0.19–1.80) 1.76 (0.96–3.23) 1.07 (0.46–2.48) 1.22 (0.76–1.95) Ref	
Child sex—male	1.24* (1.06–1.46)	10.8 (3.0, 18.7)				
**Reproductive history**						
Previous stillbirth	2.39^†^ (1.54–3.71)	1.4 (-0.7, 3.5)	1.46 (0.91–2.37)		2.47^†^ (1.52–4.00)	1.5 (-1.0, 3.9)
Previous preterm birth (<37 weeks)	1.99^†^ (1.51–2.63)	4.4 (1.1, 7.7)	2.64^†^ (1.98–3.51)	7.0 (3.2, 10.9)	1.44 (0.99–2.10)	
Previous caesarean section	1.82^†^ (1.38–2.40)	3.8 (1.1, 6.6)	1.88^†^ (1.41–2.51)	4.1 (0.8, 7.4)	2.70^†^ (1.99–3.66)	7.7 (3.9, 11.5)
Number of previous pregnancies None 1 2 3 4 or more	1.52* (1.10–2.09) 1.07 (0.79–1.46) 1.09 (0.79–1.49) 1.08 (0.78–1.49) Ref	13.9 (8.7, 19.1)	0.91 (0.65–1.27) 0.87 (0.64–1.18) 0.90 (0.66–1.22) 1.00 (0.72–1.39) Ref		0.81 (0.56–1.18) 0.62* (0.44–0.88) 0.78 (0.55–1.10) 0.82 (0.57–1.20) Ref	-13.3 (-19.0, -7.6)
**Maternal conditions**						
Essential hypertension			1.13 (0.48–2.63)		2.99^†^ (1.88–4.77)	1.0 (-1.2, 3.2)
Genital herpes	0.11* (0.02–0.73)	-1.4 (-1.7, -1.1)				
Smoking	1.18 (0.95–1.45)		0.91 (0.72–1.16)		0.77 (0.57–1.04)	
Pre-existing diabetes mellitus	2.31 (0.95–5.62)		5.06^†^ (2.43–10.55)	0.6 (-0.6, 1.8)	5.61^†^ (2.95–10.66)	0.7 (-0.6, 2.0)
Overweight/Obesity diagnosis	1.32 (0.74–2.37)		2.10* (1.20–3.66)	0.9 (-0.7, 2.5)	2.57^†^ (1.60–4.13)	1.3 (-0.8, 3.4)
Substance Abuse diagnosis	1.09 (0.79–1.52)		1.14 (0.80–1.64)		0.82 (0.52–1.31)	
Mental Health diagnosis	1.37* (1.09–1.71)	3.8 (0.3, 7.3)	1.23 (0.95–1.60)		1.34* (1.02–1.76)	3.5 (0.7, 7.7)
**Pregnancy complications**						
Threatened abortion (< 20 weeks)	2.30^†^ (1.75–3.04)	4.7 (2.2, 7.3)	3.18^†^ (2.45–4.14)	7.7 (4.3, 11.0)	1.83* (1.22–2.75)	3.0 (0.6, 5.5)
Urinary tract infection	0.94 (0.67–1.31)		0.79 (0.50–1.24)			
Pre-eclampsia					14.30^†^ (10.84–18.87)	15.4 (11.1, 19.6)
APH–placenta praevia	3.77^†^ (1.98–7.18)	0.7 (-0.5, 1.9)	3.07^†^ (1.67–5.66)	0.5 (-0.9, 2.0)	7.45^†^ (3.70–15.01)	1.6 (-0.2, 3.4)
APH–placental abruption	24.13^†^ (17.27–33.70)	5.4 (2.8, 8.0)	16.37^†^ (11.03–24.29)	3.3 (1.0, 5.6)	22.35^†^ (13.46–37.12)	4.5 (1.9, 7.2)
APH–other	9.52^†^ (7.71–11.74)	15.1 (11.6, 18.6)	11.38^†^ (9.05–14.31)	17.7 (13.7, 21.6)	2.37* (1.36–4.14)	2.7 (0.3, 5.1)
Threatened preterm labour (<37 wks)	13.24^†^ (10.60–16.54)	15.9 (12.1, 19.6)	6.40^†^ (4.87–8.41)	7.3 (3.8, 10.8)	1.52 (0.88–2.60)	
Fertility treatment	2.89^†^ (2.02–4.13)	2.7 (0.8, 4.6)	4.36^†^ (3.08–6.18)	4.7 (2.1, 7.3)	1.21 (0.66–2.24)	
Amniocentesis	2.23^†^ (1.49–3.33)	2.2 (0.7, 3.8)	2.84^†^ (1.92–4.22)	3.3 (0.9, 5.7)	6.49^†^ (4.63–9.11)	9.3 (6.0, 12.6)
Fetal growth Very small for gestation (<3%) Small for gestation (≥3% <10%) Large for gestation (>90% ≤97%) Very large for gestation (>97%) 10–90%	1.23 (0.79–1.87) 0.43^†^ (0.27–0.70) 1.58* (1.17–2.13) 1.82* (1.22–2.70) Ref	-4.2 (-5.9, -2.5) 3.4 (1.2, 5.5) 1.9 (0.4, 3.4)			11.75^†^ (8.97–15.39) 2.97^†^ (2.22–3.97) 0.26^†^ (0.12–0.57) 0.48 (0.17–1.29) Ref	22.6 (18.2, 26.9) 12.2 (8.4, 16.0) -4.6 (-5.8, -3.4)

*p < .05

^†^*p* < .001, Ref = reference category

^a^ Compared to spontaneous term births (*N* = 150,584)

The other significant risk factor for preterm pre-labour rupture of membranes extreme preterm birth was maternal age of 40 years or more. Other significant risk factors for medically indicated extreme preterm birth were essential hypertension, pre-eclampsia, being small or very small for gestational age, and being born later in the study period. In contrast, having 1 previous pregnancy was significantly protective against medically indicated extreme preterm birth as was being large for gestational age.

There were differences in the factors with the highest PAR values for each labour onset type. The categories of fetal growth restriction collectively accounted for the highest proportion of the PAR for medically indicated extreme preterm births: 22.6% (95% CI: 18.2–26.9%) for growth restriction categorised as very small for gestation, and 12.2% (95% CI: 8.4–16.0%) for small for gestation. A notable PAR was also recorded for pre-eclampsia (15.4%; 95% CI: 11.1–19.6%) with respect to medically indicated extreme preterm births. APH–other (17.7%; 95% CI: 13.7–21.6%) was the only factor with a PAR of over 10% for preterm pre-labour rupture of membranes extreme preterm births. Threatened preterm labour (15.9%; 95% CI: 12.1–19.6%), APH–other (15.1%; 95% CI: 11.6–18.6%), primigravida (13.9%; 95% CI: 8.7–19.1%), and the child being male (10.8%; 95% CI: 3.0–18.7%) accounted for the largest proportion of the PAR for spontaneous extreme preterm birth.

## Discussion

### Main findings and interpretation

The current study sought to use linked Western Australian administrative data to examine the antecedent risks to extreme preterm births, with consideration of labour onset categories and the use of PARs. Overall, factors associated with placental dysfunction (fetal growth restriction, pre-eclampsia, and antepartum haemorrhage) represent the most prominent risks to extreme preterm birth, although individual risks associated with reproductive history, maternal conditions, pregnancy complications and socio-demographic circumstances also form an important part of the risk profile. This is consistent with the findings of previous research into the risk factors for preterm birth more broadly [8]. Together these findings underscore the role of pregnancy conditions related to placental dysfunction in the aetiology of extreme preterm birth. It is also important to note that previous research has found that placental dysfunction has a high recurrence risk [32] and that this may even extend across generations [33]. Thus, research that furthers our understanding of the complex mechanisms and pathways to placental dysfunction is a major key to inform interventions aimed at decreasing the incidence of preterm birth and associated mortality and morbidity [34].

Consistent with the findings of previous preterm birth research [13–15], we also revealed nuances in the antecedent risk profiles by labour onset type. For example, factors associated with placental dysfunction typically had PARs of over 10%, although there was variation in the effect of individual factors across labour onset types: antepartum haemorrhage accounted for 21% of the PAR for spontaneous, 21% of the PAR for preterm pre-labour rupture of membranes and 9% of the PAR for medically indicated extreme preterm births, whereas fetal growth restriction and pre-eclampsia accounted for 50% of the PAR for medically indicated extreme preterm births. Previous research has identified pre-eclampsia as one of the major pathologies responsible for iatrogenic delivery in developed countries [9]. In addition, the child being male and first pregnancies were at an elevated risk of a spontaneous extreme preterm birth, but not preterm pre-labour rupture of membranes or medically indicated. These results underscore the importance of disaggregating the data by both gestational age and labour onset type when addressing risks in preterm birth.

Results revealed a significant increase in the extreme preterm birth rate over time when medical terminations and birth defects were included but no change when medical terminations and birth defects were excluded. This is consistent with our earlier work which found that changes in the medical termination rate, mainly related to improvements in the prenatal diagnosis of structural fetal anomalies, had a significant impact on the extreme preterm stillbirth rate across this period [5].

After medical terminations and birth defects were excluded, the rate of medically indicated extreme preterm births was found to significantly increase over time (however the majority of the increase in the rate of medically indicated extremely preterm births is explained by medical terminations and birth defects), whereas the rate of preterm pre-labour rupture of membranes extreme preterm births demonstrated a significant reduction. The rate of spontaneous extreme preterm births did not significantly change over time. Future research should investigate the causes of the increase in medically indicated extreme preterm births and the decrease in preterm pre-labour rupture of membranes extreme preterm births with increased antenatal surveillance and changing maternal risk profiles likely to play a role (see S1 Table).

### Strengths and limitations

A major strength of the current study is the use of linked administrative data enabling population-representative analysis that: (1) can account for medical terminations and birth defects in the examination of trends in birth rates, and (2) supports the simultaneous inclusion of a broad range of factors in examining antecedent risk profiles. However, the MNS excludes information on births that occurred before 20 weeks gestation. Because this is the period when the vast majority of medical terminations are performed [35, 36] one of the limitations of the current study is that the observed trends in extreme preterm birth are likely to have been influenced by medical terminations that occurred before 20 weeks gestation.

There are, typically, some data quality issues associated with the use of linked administrative data–these extend to the quality of linkage processes, changes in the methods of collection over time and between different sites, among others. Quality assessments consistently show that information from our source datasets are of high quality, low levels of missing information, and supported by best practice protocols that reduce missing links to negligible levels [19–21]. Also, we did not have access to information on some established risks to preterm birth, including some antenatal care events (and their timing) and pathological data that measures placental function and infection [34, 37, 38].

## Conclusions

This study is the first to find that the increase in the extreme preterm birth rate observed in a high-income jurisdiction is no longer evident after medical terminations and birth defects are excluded. The findings underscore the role of pregnancy conditions related to placental dysfunction in the aetiology of extreme preterm birth. Identifying women at risk of placental dysfunction because of personal or family history and implementing effective interventions presents the greatest opportunity to decrease the population-level impact of extreme preterm birth.

## Supporting information

S1 Table Prevalence of risk factors for all births (excluding medical terminations and birth defects), 1998–2010 (N = 603,351). (DOCX)

## References

[1] Norman JE, Morris C, Chalmers J. The Effect of Changing Patterns of Obstetric Care in Scotland (1980–2004) on Rates of Preterm Birth and Its Neonatal Consequences: Perinatal Database Study. PLoS Med. 2009;6(9):e1000153 10.1371/journal.pmed.1000153 19771156 PMC2740823

[2] Langhoff-Roos J, Kesmodel U, Jacobsson B, Rasmussen S, Vogel I. Spontaneous preterm delivery in primiparous women at low risk in Denmark: population based study. BMJ. 2006;332(7547):937–9. Epub 2006/02/25. 10.1136/bmj.38751.524132.2F 16497733 PMC1444877

[3] Tracy SK, Tracy MB, Dean J, Laws P, Sullivan E. Spontaneous preterm birth of liveborn infants in women at low risk in Australia over 10 years: a population-based study. BJOG: An International Journal of Obstetrics & Gynaecology. 2007;114(6):731–5. 10.1111/j.1471-0528.2007.01323.x 17516965

[4] Martin JA, Hamilton BE, Sutton PD, Ventura SJ, Menacker F, Kirmeyer S. Births: final data for 2004. Natl Vital Stat Rep. 2006;55(1):1–101. Epub 2006/10/21. . 17051727

[5] Farrant BM, Stanley FJ, Hardelid P, Shepherd CCJ. Stillbirth and neonatal death rates across time: the influence of pregnancy terminations and birth defects in a Western Australian population-based cohort study. BMC Pregnancy and Childbirth. 2016;16(1):1–10. 10.1186/s12884-016-0904-1 27188164 PMC4869269

[6] Lawn JE, Gravett MG, Nunes TM, Rubens CE, Stanton C. Global report on preterm birth and stillbirth (1 of 7): definitions, description of the burden and opportunities to improve data. BMC Pregnancy and Childbirth. 2010;10(1):S1 10.1186/1471-2393-10-s1-s1 20233382 PMC2841772

[7] Lumley J. Defining the problem: the epidemiology of preterm birth. BJOG: an international journal of obstetrics and gynaecology. 2003;110:3–7. 10.1046/j.1471-0528.2003.00011.x 12763104

[8] Hammond G, Langridge A, Leonard H, Hagan R, Jacoby P, DeKlerk N, et al Changes in risk factors for preterm birth in Western Australia 1984–2006. BJOG: An International Journal of Obstetrics & Gynaecology. 2013;120(9):1051–60. 10.1111/1471-0528.12188 . 23639083

[9] Steer P. The epidemiology of preterm labour. BJOG: An International Journal of Obstetrics & Gynaecology. 2005;112:1–3. 10.1111/j.1471-0528.2005.00575.x 15715585

[10] Usynina AA, Postoev VA, Grjibovski AM, Krettek A, Nieboer E, Odland JO, et al Maternal Risk Factors for Preterm Birth in Murmansk County, Russia: A Registry-Based Study. Paediatr Perinat Epidemiol. 2016;30(5):462–72. 10.1111/ppe.12304 . 27225064

[11] Khashan AS, McNamee R, Abel KM, Mortensen PB, Kenny LC, Pedersen MG, et al Rates of preterm birth following antenatal maternal exposure to severe life events: a population-based cohort study. Hum Reprod. 2009;24(2):429–37. 10.1093/humrep/den418 . 19054778

[12] Salihu H, Mbah AK, Alio AP, Kornosky JL, Whiteman VE, Belogolovkin V, et al Nulliparity and preterm birth in the era of obesity epidemic. J Matern Fetal Neonatal Med. 2010;23(12):1444–50. 10.3109/14767051003678044 . 20482286

[13] Henderson JJ, McWilliam OA, Newnham JP, Pennell CE. Preterm birth aetiology 2004–2008. Maternal factors associated with three phenotypes: spontaneous preterm labour, preterm pre-labour rupture of membranes and medically indicated preterm birth. J Matern Fetal Neonatal Med. 2012;25(6):642–7. Epub 2011/08/11. 10.3109/14767058.2011.597899 . 21827362

[14] Dekker GA, Lee SY, North RA, McCowan LM, Simpson NAB, Roberts CT. Risk Factors for Preterm Birth in an International Prospective Cohort of Nulliparous Women. PLoS ONE. 2012;7(7):e39154 10.1371/journal.pone.0039154 22815699 PMC3398037

[15] Gimenez LG, Krupitzki HB, Momany AM, Gili JA, Poletta FA, Campana H, et al Maternal and neonatal epidemiological features in clinical subtypes of preterm birth. J Matern Fetal Neonatal Med. 2016;29(19):3153–61. 10.3109/14767058.2015.1118035 . 26701680 PMC4979077

[16] Jelliffe-Pawlowski LL, Baer RJ, Blumenfeld YJ, Ryckman KK, O'Brodovich HM, Gould JB, et al Maternal characteristics and mid-pregnancy serum biomarkers as risk factors for subtypes of preterm birth. BJOG: An International Journal of Obstetrics & Gynaecology. 2015;122(11):1484–93. 10.1111/1471-0528.13495 26111589 PMC4704442

[17] Bower C, Ryan A, Rudy E. Ascertainment of pregnancies terminated because of birth defects: effect on completeness of adding a new source of data.[erratum appears in Teratology 2001 Mar;63(3):164]. Teratology. 2001;63(1):23–5. 10.1002/1096-9926(200101)63:1<23::AID-TERA1004>3.0.CO;2-S . 11169551

[18] Holman CD, Bass AJ, Rouse IL, Hobbs MS. Population-based linkage of health records in Western Australia: development of a health services research linked database. Aust N Z J Public Health. 1999;23(5):453–9. . 10575763 10.1111/j.1467-842x.1999.tb01297.x

[19] Holman CDAJ Bass JA, Rosman DL Smith MB, Semmens JB Glasson EJ, et al A decade of data linkage in Western Australia: strategic design, applications and benefits of the WA data linkage system. Aust Health Rev. 2008;32(4):766–77. 18980573 10.1071/ah080766

[20] Gee V, Dawes V. Validation study of the Western Australian Midwives' Notification System 1992. Perth: Health Department of Western Australia, 1994 Contract No.: Occasional paper 59.

[21] Downey F. Validation Study of the Western Australian Midwives’ Notification System. 2005 Data. Perth, Western Australia: 2007 Statistical series number 78 ISSN: 0816-2999.

[22] Kelman CW, Bass AJ, Holman CD. Research use of linked health data—a best practice protocol. Aust N Z J Public Health. 2002;26(3):251–5. . 12141621 10.1111/j.1467-842x.2002.tb00682.x

[23] World Health Organization. Preterm birth: WHO; 2018 [updated 19 February 2018; cited 2018 20 June 2018]. Available from: http://www.who.int/mediacentre/factsheets/fs363/en/.

[24] ABS. Census of Population and Housing: Socio-Economic Indexes for Area's (SEIFA), 2001. Canberra: Australian Bureau of Statistics. Catalogue number 2039.0.55.001; 2004.

[25] DoHWA. Guidelines for Completing Notification of Case Attended for Birth. Perth, Western Australia: Department of Health; 2014. Available from: http://www.health.wa.gov.au/publications/documents/Guidelines_for_Completion_of_NOCA.pdf.

[26] Dobbins TA, Sullivan EA, Roberts CL, Simpson JM. Australian national birthweight percentiles by sex and gestational age, 1998–2007. Med J Aust. 2012;197(5):291–4. Epub 2012/09/04. . 22938128 10.5694/mja11.11331

[27] Dickinson JE. Late pregnancy termination within a legislated medical environment. Aust N Z J Obstet Gynaecol. 2004;44(4):337–41. 10.1111/j.1479-828X.2004.00252.x 15282007

[28] Health Act 1911.

[29] Western Australian Register of Developmental Anomalies 2015 [cited 2015 10 September]. Available from: http://www.kemh.health.wa.gov.au/services/register_developmental_anomalies/diagnostic_codes_birth_defects.htm.

[30] Zou G. A modified poisson regression approach to prospective studies with binary data. Am J Epidemiol. 2004;159(7):702–6. Epub 2004/03/23. . 15033648 10.1093/aje/kwh090

[31] Hildebrandt M, Bender R, Gehrmann U, Blettner M. Calculating confidence intervals for impact numbers. BMC medical research methodology. 2006;6:32 Epub 2006/07/14. 10.1186/1471-2288-6-32 16836748 PMC1569862

[32] Lykke JA, Paidas MJ, Langhoff-Roos J. Recurring complications in second pregnancy. Obstet Gynecol. 2009;113(6):1217–24. Epub 2009/05/23. 10.1097/AOG.0b013e3181a66f2d . 19461415

[33] Wikstrom AK, Svensson T, Kieler H, Cnattingius S. Recurrence of placental dysfunction disorders across generations. Am J Obstet Gynecol. 2011;205(5):454.e1–8. 10.1016/j.ajog.2011.05.009 . 21722870

[34] Di Renzo GC, Tosto V, Giardina I. The biological basis and prevention of preterm birth. Best Pract Res Clin Obstet Gynaecol. 2018;16:16 10.1016/j.bpobgyn.2018.01.022 . 29703554

[35] Bourke J, Bower C, Blair E, Charles A, Knuiman M. The effect of terminations of pregnancy for fetal abnormalities on trends in mortality to one year of age in Western Australia. Paediatr Perinat Epidemiol. 2005;19(4):284–93. 10.1111/j.1365-3016.2005.00666.x 15958151

[36] Hutchinson M, Joyce A, Cheong M. Induced Abortions in Western Australia 2010–2012. 4th Report of the Western Australian Abortion Notification System. Western Australia: Department of Health, 2013.

[37] Audette MC, Kingdom JC. Screening for fetal growth restriction and placental insufficiency. Seminars In Fetal & Neonatal Medicine. 2018;23(2):119–25. 10.1016/j.siny.2017.11.004 . 29221766

[38] Yeoh PL, Hornetz K, Shauki NIA, Dahlui M. Evaluating the quality of antenatal care and pregnancy outcomes using content and utilization assessment. Int J Qual Health Care. 2018;30(6):466–71. 10.1093/intqhc/mzy041 . 29590356

