# Supplementary material for: Trends and predictors of extreme preterm birth: Western Australian population-based cohort study
Source: PLoS One. 2019 Mar 26;14(3):e0214445. doi: 10.1371/journal.pone.0214445 (PMC6435137; doi:10.1371/journal.pone.0214445)
Supplement: S1 Table — (DOCX) [file pone.0214445.s001.docx]

**S1 Table. Prevalence of risk factors for all births (excluding medical terminations and birth defects), 1998-2010 (N=603,351)**

|  | 1986-89  (% of births) | 1990-93  (% of births) | 1994-97  (% of births) | 1998-02  (% of births) | 2003-06  (% of births) | 2007-10  (% of births) |
| --- | --- | --- | --- | --- | --- | --- |
|  | n=91,409 | n=93,434 | n=92,817 | n=115,006 | n=96,870 | n=113,815 |
| **Sociodemographic** |  |  |  |  |  |  |
| SES (SEIFA disadvantage – quintiles) |  |  |  |  |  |  |
| 1 | 21.1 | 20.7 | 19.4 | 19.2 | 18.2 | 17.8 |
| 2 | 22.4 | 23.0 | 23.1 | 22.8 | 22.4 | 22.5 |
| 3 | 17.1 | 18.7 | 20.6 | 21.0 | 21.1 | 21.8 |
| 4 | 17.7 | 16.7 | 17.2 | 17.6 | 18.5 | 20.2 |
| 5 | 21.6 | 20.9 | 19.7 | 19.3 | 19.7 | 17.6 |
| Level of relative isolation (ARIA) |  |  |  |  |  |  |
| Extreme | 9.4 | 8.7 | 7.7 | 7.6 | 7.1 | 6.8 |
| High | 4.9 | 4.5 | 3.9 | 3.9 | 3.5 | 3.2 |
| Moderate | 9.7 | 9.9 | 10.0 | 10.1 | 9.5 | 9.2 |
| Low | 26.1 | 28.9 | 31.4 | 32.2 | 33.5 | 36.5 |
| None | 50.0 | 48.0 | 47.0 | 46.2 | 46.4 | 44.3 |
| Maternal age |  |  |  |  |  |  |
| < 20 | 6.5 | 6.4 | 6.2 | 6.0 | 5.5 | 4.9 |
| 20-24 | 23.3 | 20.9 | 19.2 | 17.0 | 15.9 | 16.0 |
| 30-34 | 23.7 | 27.3 | 28.9 | 30.5 | 32.4 | 30.6 |
| 35-39 | 7.0 | 8.8 | 11.1 | 13.4 | 16.0 | 17.5 |
| 40+ | 0.9 | 1.3 | 1.7 | 2.3 | 3.0 | 3.4 |
| 25-29 | 38.7 | 35.2 | 32.9 | 30.8 | 27.1 | 27.6 |
| Marital status |  |  |  |  |  |  |
| No | 11.7 | 10.8 | 11.1 | 10.1 | 9.0 | 11.6 |
| Married (including de facto) | 88.3 | 89.2 | 88.9 | 89.9 | 91.0 | 88.4 |
| Maternal ethnicity |  |  |  |  |  |  |
| Indigenous | .. | .. | .. | 6.5 | 6.4 | 5.8 |
| Asian | .. | .. | .. | 5.1 | 5.5 | 6.2 |
| Indian | .. | .. | .. | 0.7 | 0.8 | 1.6 |
| African | .. | .. | .. | 0.3 | 0.8 | 1.5 |
| Maori | .. | .. | .. | 0.7 | 0.8 | 1.0 |
| ‘Other’ | .. | .. | .. | 2.0 | 2.8 | 4.3 |
| Caucasian | .. | .. | .. | 84.7 | 83.0 | 79.6 |
| Child sex - male | 51.1 | 51.1 | 51.1 | 50.7 | 50.6 | 50.9 |
| **Reproductive history** |  |  |  |  |  |  |
| Previous stillbirth | 1.7 | 1.5 | 1.5 | 1.6 | 1.4 | 1.3 |
| Previous preterm birth (<37 wks) | 1.3 | 3.8 | 4.8 | 5.3 | 5.4 | 5.5 |
| Previous caesarean section | .. | 6.8 | 7.5 | 11.8 | 14.3 | 16.0 |
| Number of previous pregnancies |  |  |  |  |  |  |
| None | 29.3 | 28.5 | 28.7 | 28.7 | 29.9 | 30.7 |
| 1 | 31.0 | 30.3 | 30.4 | 30.8 | 30.8 | 30.9 |
| 2 | 20.3 | 20.3 | 19.6 | 19.3 | 18.8 | 18.4 |
| 3 | 10.5 | 10.9 | 10.7 | 10.4 | 9.7 | 9.7 |
| 4 or more | 8.9 | 10.0 | 10.6 | 10.7 | 10.8 | 10.4 |
| **Maternal conditions** |  |  |  |  |  |  |
| Essential hypertension | 0.7 | 0.8 | 0.7 | 0.9 | 0.9 | 1.2 |
| Maternal asthma | .. | 5.8 | 7.0 | 10.0 | 10.5 | 10.8 |
| Genital herpes | .. | 0.7 | 0.8 | 1.9 | 1.8 | 1.8 |
| Smoking | .. | .. | 22.0 | 21.8 | 17.7 | 14.8 |
| Pre-existing diabetes mellitus | .. | .. | 0.1 | 0.4 | 0.6 | 0.7 |
| Overweight/Obesity diagnosis | 0.6 | 0.7 | 0.9 | 1.3 | 1.4 | 2.0 |
| Substance Abuse diagnosis | 1.9 | 3.2 | 4.2 | 4.4 | 4.2 | 3.8 |
| Mental Health diagnosis | 5.8 | 7.2 | 8.6 | 10.7 | 12.1 | 12.2 |
| **Pregnancy complications** |  |  |  |  |  |  |
| Threatened abortion (<20 wks) | 4.2 | 5.5 | 5.8 | 5.4 | 4.8 | 3.2 |
| Urinary tract infection | 4.0 | 4.6 | 5.4 | 3.9 | 3.6 | 3.2 |
| Pre-eclampsia | 6.9 | 6.1 | 7.1 | 5.7 | 3.8 | 2.6 |
| APH – placenta praevia | 0.7 | 0.7 | 0.6 | 0.7 | 0.7 | 0.7 |
| APH – placental abruption | 0.8 | 0.7 | 0.6 | 0.6 | 0.4 | 0.3 |
| APH – other | 2.4 | 2.9 | 2.9 | 2.6 | 2.8 | 2.4 |
| Threatened preterm labour (<37 wks) | .. | 1.1 | 1.3 | 2.5 | 2.3 | 2.2 |
| Fertility treatment | .. | 1.1 | 1.1 | 1.6 | 2.1 | 2.6 |
| CVS/placental biopsy | .. | 0.6 | 0.4 | 0.4 | 0.3 | 0.3 |
| Amniocentesis | .. | 3.7 | 4.7 | 3.8 | 2.2 | 1.5 |
| Fetal growth |  |  |  |  |  |  |
| Very small for gestation (<3%) | 3.5 | 3.4 | 3.2 | 2.7 | 2.5 | 2.5 |
| Small for gestation (≥3% <10%) | 8.1 | 7.8 | 7.6 | 6.8 | 6.6 | 6.1 |
| Large for gestation (>90% ≤97%) | 5.6 | 5.8 | 6.1 | 6.5 | 7.0 | 7.2 |
| Very large for gestation (>97%) | 2.3 | 2.4 | 2.5 | 3.0 | 3.1 | 3.2 |
| 10-90% | 80.6 | 80.7 | 80.7 | 81.0 | 80.9 | 81.0 |
